# Smartphone Use Among University Students During COVID-19 Quarantine: An Ethical Trigger

**DOI:** 10.3389/fpubh.2021.600134

**Published:** 2021-07-26

**Authors:** Heba Saadeh, Reem Q. Al Fayez, Assem Al Refaei, Nour Shewaikani, Hamzah Khawaldah, Sobuh Abu-Shanab, Maysa Al-Hussaini

**Affiliations:** ^1^Department of Computer Science, King Abdullah II School of Information Technology Faculty, The University of Jordan, Amman, Jordan; ^2^Department of Computer Information System, King Abdullah II School of Information Technology Faculty, The University of Jordan, Amman, Jordan; ^3^School of Medicine, The University of Jordan, Amman, Jordan; ^4^Department of Geography, School of Arts, The University of Jordan, Amman, Jordan; ^5^Psychosocial Program, King Hussein Cancer Center, Amman, Jordan; ^6^Department of Pathology and Laboratory Medicine, King Hussein Cancer Center, Amman, Jordan; ^7^Human Research Protection Program Office, King Hussein Cancer Center, Amman, Jordan

**Keywords:** COVID-19, Jordan, quarantine, short version addiction scale, smartphone addiction, university students

## Abstract

To reduce the spread of COVID-19, Jordan enforced 10 weeks of home quarantine in the spring of 2020. A cross-sectional study was designed to assess this extended quarantine's effect on smartphone addiction levels among undergraduates. A random sample of 6,157 undergraduates completed an online questionnaire (mean age 19.79 ± 1.67 years; males 28.7%). The questionnaire contains different sections to collect socio-demographic, socio-economic, academic, quarantine-related information, and smartphone usage. The smartphone addiction scale-short version was used to assess the degree of addiction during the quarantine. The mean addiction score across the whole sample was 35.66 ± 12.08, while the prevalence of addiction among participants was 62.4% (63.5% in males and 61.9% in females). The majority of the participants (85%) reported that their smartphone usage during the quarantine increased or greatly increased (27.6 and 57.2%, respectively), with some 42% using their smartphones for more than 6 h a day. Nevertheless, three-quarters of the students wished to reduce their smartphone usage. Several demographic and quarantine factors have been assessed, and students' gender, the field of study, parental education, household income in addition to the location of quarantine (urban, rural) and the house specifications (apartment, independent house, with/without a garden) showed statistically significant associations with smartphone addiction during the quarantine. Female students, students studying scientific- and medical-related majors compared to those studying humanity majors, those with higher incomes, those who had been quarantined in an apartment without a garden, and those who lived in urban areas showed significantly higher addiction scores.

## Introduction

The novel coronavirus 2019 (COVID-19) infected more than 180 million people in 222 countries and killed around 4 million globally (as of 07/07/2021), according to the World Health Organization (1, 2). This disease is a severe acute respiratory syndrome caused by betacoronavirus (SARS-CoV-2), which might disrupt the human body's normal immune response and cause lots of implications (3, 4). Therefore, the vagaries of this pandemic forced many countries to take severe actions to protect their citizens from infection. Jordan applied complete lockdown around mid-March 2020, closing all schools, universities, shops, public and private sectors, borders, and airlines, forbidding any civil movement for several days. A curfew was then applied to restrict all movement, allowing only short walks and for short periods. The majority of the population was under home quarantine for around 10 weeks. These extreme measures helped contain the spread of the virus and controlled the number of casualties and deaths in Jordan in the spring and summer of 2020. Due to the countrywide closure, schools, universities, and companies moved to online platforms for distance learning and remote working. This new lifestyle, enforced by staying at home and under quarantine, has brought new challenges socially, economically, physiologically, and psychologically (5–9).

One significant lifestyle shift is the complete reliance on the internet and smart devices, like tablets, laptops, and mobiles. During the quarantine, with the necessary social/spatial distancing, the usage of these smart devices increased at an increasingly fast pace. Unfortunately, this total dependence has shown to be a form of addiction, i.e., a compulsive physiological need for and use of a habit-forming substance (10). Nowadays, addiction is not only restricted to extensive substance or drug abuse but also extends to the behavioral obsession with a specific activity that disturbs people's healthy daily lives. Recently, internet-based activities, like online gaming, chatting, and communications through the different available applications, have shown similar addiction levels to those of drugs (11–13).

The impact of internet misusage has increased significantly due to its high accessibility through smartphones, especially during the COVID-19 pandemic. Mobile phones are widely used; around 60% of the world's population and 80% of Jordanian households have mobiles (14, 15). In the past year alone, Jordanian mobile phone connections, internet users, and active social media users increased by 1.7, 1.2, and 7.4%, respectively (15). Several studies have identified the prevalence of smartphone addiction risks in different countries, using the smartphone addiction scale-short version (SAS-SV) (16–25). Although a few recent studies have highlighted the different aspects of internet usage related to COVID-19 (26–29), none, to the best of our knowledge, have examined smartphone addiction during the current lockdown and quarantine. This is the first research that presents a large-scale study of thousands of Jordanian undergraduate students to assess the effect of COVID-19 extended home quarantine on smartphone addiction levels. This is assessed by collecting many exposures to cover the demographic, economic, and quarantine-related factors that might worsen the effect of quarantine on smartphone overuse.

## Methods

### Participants

Responses to the online questionnaire were submitted by 7,146 undergraduates at the University of Jordan (UJ) during the April and May of 2020. After cleaning the data by removing all duplications, 6,157 unique participants who had fully completed the online questionnaire and participated voluntarily remained for analysis. There was no missing data as all the questions were mandatory. Participants could withdraw at any time by failing to answer any of the questions. The study's purpose and procedures had been approved by the Institutional Review Board and the Research Ethics Committee at UJ.

Participants' ages ranged between 17 and 30 years, with a mean of 19.79 ± 1.67. 1,769 students were male (28.7%) and 4,388 female (71.3%). Half were studying humanities-related majors and around one-third scientific majors, with the rest studying medical-related majors. Nearly half of the students were in their first year.

### Measurements

This study focuses on the association between the new lifestyle forced by home quarantine and smartphone usage, which might even reach the addiction level. The online questionnaire was distributed in Arabic, the Arabic version of the SAS-SV was validated in 2018 (30), targeting all UJ undergraduates and ensuring that all the participants fully understood the questions and the accompanying choices. The questionnaire contains several sections, collecting an extensive list of exposures, like socio-demographic, socio-economic, and quarantine-related information, in addition to the 10 items of the SAS-SV to measure the primary outcome: smartphone addiction level. Strengthening the Reporting of Observational Studies in Epidemiology (STROBE) statement guidelines for observational cross-sectional studies were used to guide the reporting of this study (31).

#### Socio-Demographic/Socio-Economic Variables

The study examined the participants' different socio-demographic measures: gender, age, place of residence, class (year at university), academic major (Scientific, Medical, or Humanities), academic performance ranging from acceptable to excellent, and their smoking practices. The study also collected a few socio-economic factors, such as parental education levels, parental employment status, and household income level ranging from <200 JD ($282) to more than 1,500 JD ($2,115).

#### Quarantine Variables

To assess the association between smartphone addiction and quarantine, 12 questions were listed. Some questions asked about the place of residence during quarantine, whether in a city or a village and the house specifications, like an apartment or a house with or without a garden. The study also asked about the number of people quarantined with each student, ranging from 0 to >10, how many children are among them, and whether they have specific health issues, including chronic diseases. The students were also asked about communication with the family members who lived with them and those who did not. Furthermore, questions about students' hobbies, including newly practiced ones started during the quarantine, and the household income during quarantine, whether it remained the same, increased, decreased, or stopped altogether, were included.

#### Smartphone Usage

##### Smartphone Addiction Scale–Short Version

The original scale consisted of 33 items developed by Kwon et al. (29). The same authors developed the short version (SAS-SV) scale in 2013 (25) to evaluate smartphone addiction's level of risk and its prevalence, based on self-reporting. It has been validated and has greater durable internal consistency than the original version (25). It has 10 items (listed in the results section), each rated on a six-point Likert scale, ranging from strongly disagree (scores 1 point) to strongly agree (scores 6 points). A high score indicates high risk but does not diagnose an addiction. According to Kwon et al. (25), different cut-off values were suggested for each gender: 31 for males and 33 for females. In this study, the short version scale was used to reduce the number of questions the participants needed to answer.

##### Usage of the Smartphone During Home Quarantine

In addition to the addiction scale, the questionnaire included a few questions regarding the number of hours spent using smartphones per day. Students were also asked about the most frequent smartphone applications (Facebook, YouTube, Twitter, Snapchat, Instagram, and Netflix) used before and during the quarantine, and the level of change in usage was assessed.

### Statistical Analysis

Descriptive statistics were performed on the whole sample. Numerical and categorical variables were summarized as mean ± standard deviation and total numbers (percentages), respectively. Binary factors were tested for significance using a two-sample *t*-test, while factors with more than two values were analyzed using a one-way analysis of variance (ANOVA). Tukey Honestly Significance Difference (TukeyHSD) was used as a *post-hoc* analysis to follow up on the significant factors that resulted from the ANOVA to identify the pair of values that had a significant mean difference. The significant factors were further investigated using logistic regression to identify the significant predictors of the addiction state, and to control the potential confounding factors and selection bias. A threshold value of *p* = 0.05 was used to test for significance. All statistical analyses were performed using R version 4.0.0 and RStudio version 1.2.5042.

## Results

The original sample consisted of 7,146 submissions, which were then reduced to 6,157 after omitting duplicated responses. Half of the 6,157 undergraduate students were in their first year; the average age was 19.79 ± 1.67. Around 70% (*n* = 4,388) were female, nearly half (*n* = 3,092) were studying humanities-related majors, and about 85% (*n* = 5,151) were non-smokers. The academic performance of the students was categorized into four levels: excellent (10.6%, *n* = 655), very good (33.5%, *n* = 2,065), good (33.4%, *n* = 2,057), and acceptable (22.5%, *n* = 1,380), as declared by the students themselves. The household income level of the participants ranged from <200 JD (1 *JD* = ~1.4 USD) to more than 1,500 JD; around 45% (*n* = 2,807) had very low to low income (<600 JD), around 30% (*n* = 1,906) had medium-level (600–1,000 JD), and the rest (*n* = 1,444) had high-level (more than a 1,000 JD) income. The majority of the students (77.2%, *n* = 4,751) lived in the capital city (Amman). Table 1 summarizes participants' demographics.

**Table 1:** Socio-demographic, socio-economic, and quarantine characteristics of study participants.

**Variable**	**Mean ± SD or *N* (N %)**	**Variable**	**Mean ± SD or *N* (N %)**
**Gender**		Age	19.79 ± 1.67
Male	1,769 (28.7%)	**Employment status (parents)**	
Female	4,388 (71.3%)	Both work	1,075 (17.5%)
**Major**		Only father works	3,762 (61.1%)
Humanities	3,092 (50.2%)	Only mother works	244 (4.0%)
Medical	840 (13.6%)	Neither work	1,076 (17.4%)
Scientific	2,235 (36.2%)	**Household Income Level**	
**Class**		Less than 200 JD	375 (6.2%)
Year 1	3,003 (48.8%)	200–400 JD	1,225 (19.9%)
Year 2	1,757 (28.5%)	400–600 JD	1,207 (19.6%)
Year 3	793 (12.9%)	600–800 JD	951 (15.4%)
Year 4	481 (7.8%)	800–1,000 JD	955 (15.5%)
> Year 4 (Year 5, Year 6, and more)	123 (2.0%)	1,000–1,200 JD	493 (8.0%)
**GPA Level**		1,200–1,500 JD	341 (5.5%)
Excellent	655 (10.6%)	More than 1,500 JD	610 (9.9%)
Very good	2,065 (33.5%)	**About to graduate**	
Good	2,057 (33.4%)	Yes	217 (3.5%)
Acceptable	1,380 (22.5%)	No	5,940 (96.5%)
**Education level (father)**		**Cigarette smoking**	
Post graduates	732 (11.9%)	Yes	1,006 (16.3%)
Bachelor	2,066 (33.6%)	No	5,151 (83.7%)
Diploma	1,126 (18.3%)	**Education level (mother)**	
High School	1,485 (24.1%)	Post Graduates	308 (5.0%)
Others (did not reach high school)	748 (12.1)	Bachelor	1,779 (28.8%)
**Location of the house during the quarantine**		Diploma	1,543 (25.1%)
Urban areas	5,315 (86.3%)	High School	1,900 (30.9%)
Rural areas	842 (13.7%)	others (did not reach high school)	627 (10.2%)
**Home specification**		**Household income during the quarantine**
Apartment with garden	1,176 (19.1%)	Increased	275 (4.5%)
Apartment without a garden	2,174 (35.3%)	Stay the same	2,640 (42.9%)
House with garden	2,210 (35.9%)	Decreased	2,467 (40.1)
House without a garden	597 (9.7%)	Stopped completely	775 (12.5)

*Total number of participants: 6,157 students*.

*SD, Standard Deviation*.

*Only age is a continuous variable. Thus, it has a mean and standard deviation, while the rest are discrete variables; therefore, they were summarized using percentages*.

Parental employment status showed that for more than half of the students (61.1%, *n* = 3,762) only their father worked and 4%(*n* = 244) only their mother; 17.5% (*n* = 1,075) had both parents working, and a similar percentage (*n* = 1,076) neither. For about one-third (*n* = 2,066) of the students, their father was educated to bachelor level, and for a similar proportion (*n* = 1,900) their mother to high school level; only around 12% (*n* = 732) and 5% (*n* = 308) of the students had fathers and mothers educated to postgraduate level, respectively (Table 1).

Around one-seventh *(*n = 842) of the students lived in a village during the quarantine. An equal proportion (*n* = 2,174 and 2,210, ~35%) lived either in an apartment without a garden or in a house with a garden, with 55% (*n* = 3,386) living in a household with a garden. For nearly 50% (*n* = 3,242) of the students, their household income either decreased or completely stopped during the quarantine, indicating financial difficulties (Table 1).

**Figure 1:**
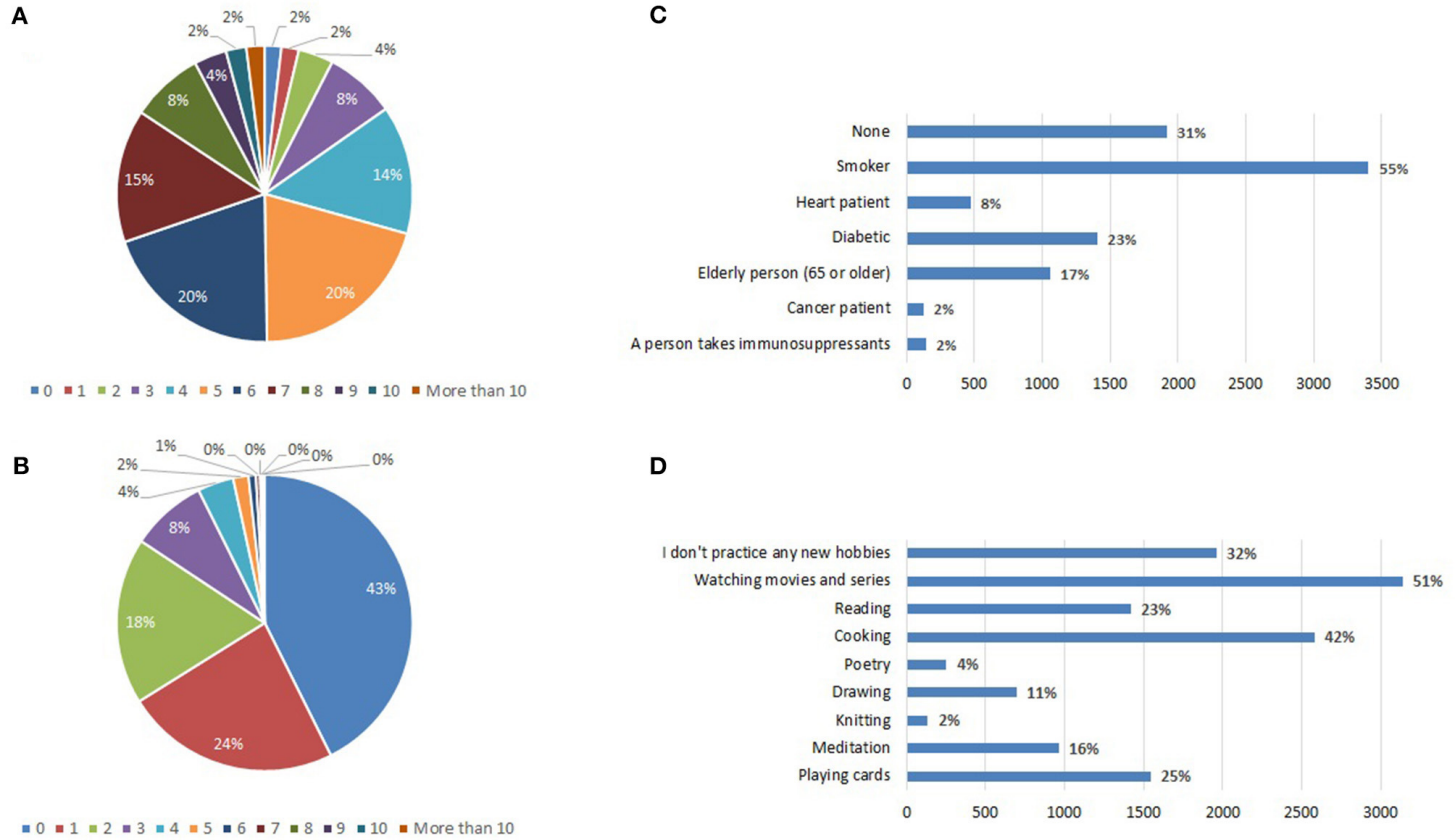
**(A)** Pie chart for the percentages of people quarantined with each of the study participants ranged from no one (0) to more than 10. **(B)** Similar to **(A)** but for the percentage of the children quarantined with each student. **(C)** Horizontal bar chart for the percentage of the quarantined people (with each student) with specific health issues. **(D)** Similar to **(C)** but for the percentages of the new hobbies that the students started to practice during the quarantine. *X*-axes in **(C)** and in **(D)** shows the frequency of the participants.

During the quarantine, 77% (*n* = 4,741) of the students lived with 3–7 family members, and 43% (*n* = 2,648) were not quarantined with children (Figures 1A,B). More than half (*n* = 3,386) were quarantined with a smoker, about 20% (*n* = 1,416) with a diabetic patient, around 8% (*n* = 493) with a cardiac patient, and 17% (*n* = 1,047) with an elderly member of the family (>65 years) (Figure 1C). The majority of the students (89.7%, *n* = 5,523) increased communication with their families, and about 70% (*n* = 4,310) communicated more with a distant family member during the quarantine. Around 80% (*n* = 4,926) spent more time with their families than they normally do.

Nearly 70% (*n* = 4,310) of the students spent most of their time watching movies/series and/or sleeping and about 50% (*n* = 3,079) in eating/cooking. Many students (68%, *n* = 4,187) started new hobbies during quarantine (Figure 1D), including watching movies/series (51%, *n* = 3,140), cooking (42%, *n* = 2,586), board games (25%, *n* = 1,539), reading (23%, *n* = 1,416), meditation (16%, *n* = 985) and drawing (11%, *n* = 677).

The primary outcome (addiction level) was assessed by the SAS-SV. The 10 items in the SAS-SV are included in Table 2. Mean scores ranged from 3.21 (item 6: Table 2) to 4.02 (item 4: Table 2). The 10 items had totals ranging between 10 (all items scored 1) and 60 (all items scored 6) with a mean score of 35.66 ± 12.08. The associations between the different demographic and quarantine variables with smartphone addiction levels, i.e., SAS-SV scores, are presented in Table 3. The lowest SAS-SV score was 33.51 ± 13.25 (house without a garden: Table 3), and the highest 36.83 ± 11.63 (apartment without a garden: Table 3). The prevalence of addiction among participants was 62.4% (*n* = 3,841), representing potential excessive use, with a mean SAS-SV score of 43.18 and a standard deviation of 7.59. However, based on the suggested SAS-SV score threshold of ≥31 for males and ≥33 for females (25), the prevalence of addiction was 63.5% (*n* = 1,124, total number of males = 1,769) and 61.9% (*n* = 2,717, total number of females 4,388) with SAS-SV scores of 42.33 ± 7.85 and 43.53 ± 7.45 for males and females, respectively (Table 4).

**Table 2:** Items of smartphone addiction scale–short version.

**Item**		**Mean ± SD**
1	Missing planned work due to smartphone use	3.71 ± 1.48
2	Having a hard time concentrating in class, while doing assignments, or while working due to smartphone use	3.68 ± 1.45
3	Feeling pain in the wrists or at the back of the neck while using a smartphone	3.61 ± 1.52
4	Will not be able to stand not having a smartphone	4.02 ± 1.57
5	Feeling impatient and fretful when I am not holding my smartphone	3.32 ± 1.54
6	Having my smartphone in my mind even when I am not using it	3.21 ± 1.53
7	I will never give up using my smartphone even when my daily life is already greatly affected by it.	3.66 ± 1.55
8	Constantly checking my smartphone so as not to miss conversations between other people on Twitter or Facebook	3.54 ± 1.53
9	Using my smartphone longer than I had intended	3.63 ± 1.53
10	The people around me tell me that I use my smartphone too much.	3.28 ± 1.53

*SD, standard deviation*.

*This scale was proposed by Kwon, Kim, Cho and Yang, 2013 (25) as a short version for the original Smartphone Addiction Scale that contained 33 items (32)*.

**Table 3:** Association between smartphone addiction level (SAS-SV score) and the socio-demographic, socio-economic and quarantine characteristics of the participants.

**Variable**	**SAS-SV score mean ± SD or**	**Variable**	**SAS-SV score mean ± SD or**
	**(*p*-value)**		**(*p*-value)**
**Gender**	**(1.4e-03**^a^^*^)	**Age**	35.66 ± 12.08
Male	34.88 ± 12.24	**Employment Status (Parents)**	(0.065^b^)
Female	35.98 ± 12.00	Both of them work	36.20 ± 12.13
**Major**	**(1.1e-03**^b^^*^)	Only Father works	35.34 ± 12.04
Humanities	35.11 ± 12.31	Only Mother works	36.58 ± 11.96
Medical	36.52 ± 12.11	None of them work	36.03 ± 12.15
Scientific	36.10 ± 11.70	**Household Income Level**	**(6.9e-04**^b^^*^)
**Class**	(0.458^b^)	Less than 200 JD	33.82 ± 12.64
Year 1	35.83 ± 12.02	200–400 JD	34.59 ± 12.56
Year 2	35.69 ± 12.18	400–600 JD	36.12 ± 12.04
Year 3	35.07 ± 12.34	600–800 JD	36.13 ± 11.85
Year 4	35.91 ± 11.53	800–1,000 JD	35.92 ± 11.92
> Year 4 (Year 5, Year 6, and more)	33.89 ± 12.40	1,000–1,200 JD	36.46 ± 11.15
**GPA Level**	(0.110 ^b^)	1,200–1,500 JD	36.13 ± 11.41
Excellent	35.74 ± 12.63	More than 1,500 JD	35.98 ± 12.33
Very Good	36.12 ± 11.95	**About to graduate**	(0.577 ^a^)
Good	35.20 ± 11.96	Yes	36.09 ± 11.56
Acceptable	35.62 ± 12.17	No	35.64 ± 12.10
**Education Level (Father)**	**(0.013** ^**b**^ ^*^)	**Cigarette Smoking**	(0.212 ^a^)
Post Graduates	35.27 ± 11.98	Yes	35.23 ± 12.08
Bachelor	35.71 ± 11.95	No	35.75 ± 12.08
Diploma	36.33 ± 11.59	**Education Level (Mother)**	**(0.030** ^**b**^ *)
High School	35.90 ± 12.38	Post Graduates	36.08 ± 11.54
Others (did not reach high school)	34.43 ± 12.57	Bachelor	35.93 ± 12.06
**Location of the house during the quarantine**	**1.9e-06** ^**a**^ *	Diploma	36.18 ± 11.92
Urban areas	35.96 ± 11.97	High School	35.25 ± 12.06
Rural areas	33.74 ± 12.59	others (did not reach high school)	34.66 ± 12.76
**Home specification**	**2.9e-10** ^**b**^ *	**Household income during the quarantine**	0.184 ^b^
Apartment with garden	35.92 ± 11.63	Increased	34.58 ± 12.57
Apartment without a garden	36.83 ± 11.63	Stay the same	35.54 ± 11.96
House with garden	34.96 ± 12.30	Decreased	36.00 ± 12.11
House without a garden	33.51 ± 13.25	Stopped completely	35.38 ± 12.20

*Total number of participants: 6,157 students; SAS-SV, Smartphone Addiction Scale Short-Version (25); SD, Standard Deviation;*

a *P-value is obtained using t-test;*

b *P-value is obtained using one-way-ANOVA*.

* *Statistically significant p-value (≤0.05). Statistically significant values appear in Bold*.

**Table 4:** Smartphone addiction prevalence among the study participants based on SAS-SV scores.

**Participant groups**	**Factor**	**Prevalence as *N* (N %)**	**SAS-SV score mean ± SD**
Potential High-risk	Male	1,124 (63.5%)	42.33 ± 7.85
	Female	2,717 (61.9%)	43.53 ± 7.45
	Total	3,841 (62.4%)	43.18 ± 7.59
Potential low-risk	Male	645 (36.5%)	21.89 ± 6.18
	Female	1,671 (38.1%)	23.69 ± 6.63
	Total	2,316 (37.6)	23.19 ± 6.56

*Total number of participants: 6,157 students: 1,769 males, and 4,388 females; SAS-SV, Smartphone Addiction Scale Short-Version (25); SD, Standard Deviation; Males cut-off is 31, and Females cut-off is 33, according to (25)*.

Among the tested binary variables, including the gender, graduation status, smoking habit, and the house location during the quarantine, both the graduation status and smoking habit variables were not significant (*p* > 0.05). Females and quarantine in urban areas were significantly associated with smartphone addiction (Table 3). Furthermore, the field of study (major), city, household income, parental education, and the house specifications were found significant (ANOVA *p* < 0.05). The mean difference between the humanities and each of the scientific and medical majors was significant (TukeyHSD *p*-values: 0.009 and 0.007, respectively), with the scientific and medical majors having a larger SAS-SV mean score than the humanities-related majors (Table 3). Although the mother's education had a significant association (ANOVA *p*-value: 0.030), the TukeyHSD analysis did not find any significant pair-wise comparison between its different values (*p* > 0.05); hence, this factor is not considered significantly associated with smartphone addiction levels. On the other hand, the household income had a significant association (ANOVA *p*-value: 6.9e-4), and this is mainly due to the difference between <200 JD and higher income levels (TukeyHSD *p* < 0.05). Likewise, the father's education (ANOVA *p*-value 0.013); only the comparison between a diploma and below high school was significant (TukeyHSD *p*-value: 0.007), while other education levels showed no significant associations (Table 3). Finally, house specifications were found to be significantly associated with addiction levels. Living in a house and not in an apartment, as well as having a garden, had lower SAS-SV scores. Quarantine in an apartment without a garden showed a higher significant association with smartphone addiction and the highest SAS-SV score (Table 3).

The six significant factors (ANOVA and TukeyHSD *p* < 0.05) of gender, house location, major, household income, father's education, and the house specifications were further investigated using logistic regression. The aim was to identify which of these factors was a significant potential predictor of the students' addiction state [calculated based on the suggested SAS-SV score thresholds of ≥31 for males and ≥33 for females (25)] (Table 5). As expected, quarantine in urban areas and studying health- or science-related majors had a significant positive association with addiction state. Quarantine in a house without a garden showed a significant negative association, indicating a SAS-SV score lower than other values, as listed in Table 3.

**Table 5:** Association between addiction state and each of the identified significant factors, as assessed by logistic regression^+^.

**Coefficients**	**Estimate**	***p*-value**	**Odd ratio**	**CI lower**	**CI upper**
(Intercept)	0.092	0.549	1.096	−0.208	0.393
Sex (Male)	0.037	0.539	1.038	−0.082	0.157
House Location (Urban)	0.215	0.010 ^*^	1.240	0.051	0.380
Specialization (Medical)	0.192	0.019 ^*^	1.212	0.032	0.354
Specialization (Scientific)	0.161	0.007 ^*^	1.175	0.045	0.278
Home specification (Apart. without a garden)	0.115	0.134	1.122	−0.036	0.264
Home specification (House with garden)	−0.122	0.119	0.885	−0.277	0.032
Home specification (House without a garden)	−0.303	0.004 ^*^	0.738	−0.508	−0.098
Household income (1,000–1,200 JD)	0.242	0.098	1.274	−0.045	0.530
Household income (1,200–1,500 JD)	0.246	0.125	1.279	−0.068	0.562
Household income (200–400 JD)	−0.051	0.670	0.950	−0.289	0.185
Household income (400–600 JD)	0.119	0.327	1.127	−0.121	0.359
Household income (600–800 JD)	0.173	0.177	1.188	−0.079	0.422
Household income (800–1,000 JD)	0.121	0.349	1.128	−0.133	0.373
Household income (>1,500 JD)	0.176	0.218	1.193	−0.104	0.456
Father education level (Diploma)	0.187	0.018^*^	1.205	0.032	0.343
Father education level (High school)	0.124	0.097	1.132	−0.022	0.271
Father education level (Post graduates)	0.0005	0.995	1.001	−0.176	0.179
Father education level (Others)	−0.012	0.895	0.988	−0.195	0.171

*CI: 95% Confidence Interval*.

* *Statistically significant p-value (≤0.05)*.

+ *Dependent variable: addiction state; calculated based on the suggested SAS-SV scores threshold of ≥31 for males and ≥ 33 for females*.

Around 85% (*n* = 5,234) of the students reported increased smartphone usage during quarantine, and only about 3% (*n* = 196) reduced their smartphone usage, which correlates well with the SAS-SV scores (Table 6). During this quarantine, around 42% (*n* = 2,575) of the students, despite their demographics, spent more than 6 h a day on their smartphones with very high SAS-SV scores (38.60 ± 11.18 for 6–8 h and 39.41 ± 13.23 for >8 h: Table 6). Only 3.6% (*n* = 223) of the students used their smartphones less than an hour per day, and they had relatively small SAS-SV scores (Table 6).

**Table 6:** Smartphone usage during quarantine and their associations with smartphone addiction level (SAS-SV score).

**Variable**	***N* (N %)**	**SAS-SV score mean ± SD**
**Smartphone usage during the quarantine**
Largely decreased	63 (1%)	26.13 ± 12.67
Decreased	133 (2.2%)	29.62 ± 11.53
Stayed the same	737 (12.0%)	29.47 ± 10.63
Increased	1,699 (27.6%)	33.85 ± 9.84
Largely increased	3,525 (57.2%)	38.23 ± 12.56
**Number of hours used on Smartphone during the quarantine**
0–0.5	95 (1.5%)	23.37 ± 12.06
0.5–1	128 (2.1%)	26.31 ± 9.45
1–2	328 (5.3%)	29.58 ± 10.88
2–3	617 (10.0%)	30.34 ± 10.39
3–4	1,008 (16.4%)	33.13 ± 10.60
4–6	1,406 (22.8%)	36.73 ± 11.00
6–8	1,178 (19.1%)	38.60 ± 11.18
Greater than 8	1,397 (22.8%)	39.41 ± 13.23

*Total number of participants: 6,157 students*.

*SAS-SV, Smartphone Addiction Scale Short-Version (25); SD, Standard Deviation*.

**Figure 2:**
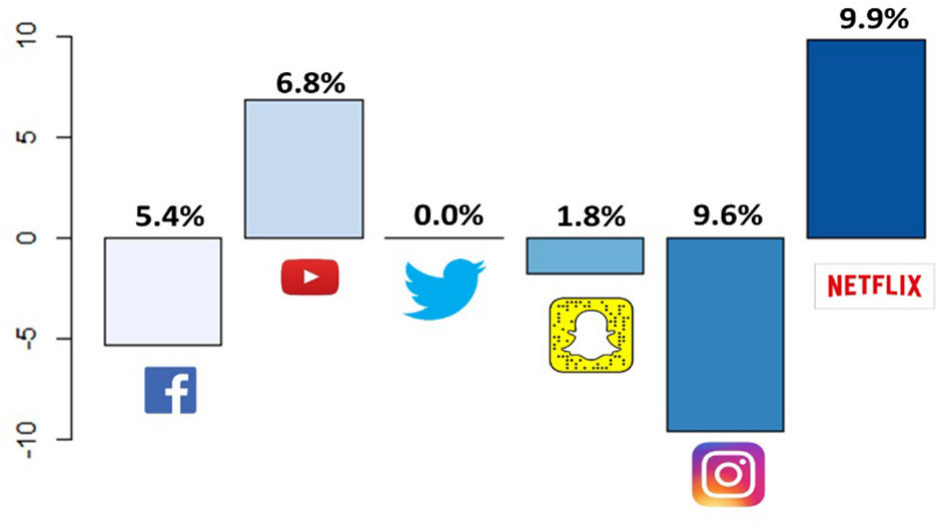
Absolute difference of smartphone applications usage (in percentages) before and during the quarantine.

The top three applications widely used on smartphones before the quarantine were reported to be Facebook and its messenger, Instagram, then YouTube. These applications remained the top three applications used by the students during the quarantine (Table 7). However, the use of Facebook and Instagram was reduced by 5.4 and 9.6%, respectively, and the use of YouTube and Netflix increased by 6.8 and 9.9%, respectively (Figure 2 and Table 7).

**Table 7:** Top smartphone applications used by the students before and during the quarantine.

**Smartphone applications**	**Usage before quarantine % (A)**	**Usage during quarantine % (B)**	**Difference between the usage before and during the quarantine (B–A)**
Facebook	45.0%	39.6%	−5.4%
Instagram	31.4%	21.8%	−9.6%
YouTube	13.2%	20.0%	6.8%
Snapchat	6.2%	4.4%	−1.8%
Twitter	2.1%	2.2%	0.0%
Netflix	2.0%	11.9%	9.9%

*Total number of participants: 6,157 students*.

Finally, around three-quarters (*n* = 4,690) of the students self-reported that they wished to change their smartphone usage by reducing the number of hours they spent using them. Only 3.4% (*n* = 208) wished to increase their usage, and around 20% (1,259) reported that they were satisfied with their current smartphone usage.

## Discussion

The study was conducted on students at the University of Jordan, the largest public university in the capital, Amman (only 22.8% of the study participants lived outside the capital). UJ hosts around 50,000 students studying undergraduate and postgraduate degrees in humanities, science, and health disciplines. Six thousand one hundred fifty-seven undergraduates voluntarily completed the online questionnaire, comprising around 12.3% of UJ students. This sample of participants is a good representative of the demographics of the university since 76% of the UJ students are female, 50.3% are studying humanities-related majors, 10.5% have excellent GPA, and 22.5% have acceptable GPA; the figures for the study participants are 71.3% females, 50.2% studying humanities, and 10.6 and 22.5% with excellent and acceptable GPAs, respectively. The questionnaire link was uploaded with several obligatory university requirements, usually taken by students in their first 2 years, thus explaining why around 77% of the participants were in years 1 and 2, with a mean age of 20 years; only 3.5% were in their final semester (Table 1).

This sample is also comparable with the Jordanian population, according to the National Council for Family Affairs (NCFA) national survey in 2017 (33). About 78% of the participating families had 3–7 members, consistent with our sample demographics (Figure 1A). Furthermore, 54.4 and 45.6% of the students lived in an apartment or individual house, respectively (Table 1), which is also similar to the corresponding NCFA survey results of 57 and 42%. The NCFA reported that 19% of female adults in Jordanian families work, a similar percentage to the 21.5% of students whose mothers worked. Non-communicable chronic diseases prevail in society as 14.5 and 7.2% suffer from diabetes and cardiovascular diseases, respectively. 23 and 8% of the students in this sample were quarantined with a family member suffering from diabetes or cardiovascular diseases, respectively (Figure 1C). Tobacco smoking in Jordan, as reported by WHO (34), is more prevalent in males, with 70% of males aged more than 14 years being smokers (35); this explains the high proportion, nearly half, of the students quarantined with a smoker (Figure 1C). The preponderance of females in our sample might account for only 16.3% being smokers.

Regrettably, around 50% of parents in our sample had partially or entirely lost their jobs, reducing financial resources since the private sector was primarily affected by the countrywide closure (Table 1). Previous studies disagreed with the effect of household income on smartphone addiction (36, 37), while in this study, lower incomes were negatively associated with addiction scores (Table 3). Similarly, contradictory results were reported regarding parental education and phone usage (36, 38). Nevertheless, this study reported a significant association between parental education and smartphone addiction, precisely the difference between a diploma and below high school (Table 3).

Interestingly, the characteristics of a quarantine site had a significant effect on smartphone usage. Most of the Jordanian population lives in urban areas; hence, a small proportion of the participants were quarantined in rural areas (13.7%: Table 1). Quarantine in an apartment without a garden was significantly associated with addiction scores (Table 3). Additionally, a significant association between quarantine in urban areas and addiction scores is noted (Tables 3, 5). This can be explained by the tight surveillance and strict control the government imposed on the big cities compared to the rural areas, which provided fewer opportunities for practicing outdoor activities and encouraged spending more time on smartphones.

The COVID-19 pandemic home quarantine enforced a sudden and different lifestyle, an extended lockdown with strict rules for remaining indoors. About 70% of the students spent most of their time watching movies/series (Figure 1D). 85% reported an increase in smartphone usage, with about 42% spending more than 6 h a day on their smartphones. Additionally, with the limited available resources within families, many students relied on their mobile phones to attend the university's compulsory online teaching.

Several studies have assessed smartphone addiction among university students; however, none have evaluated its addiction and prevalence during a quarantine. SAS-SV results indicated that smartphone addiction was prevalent in a total of 3,841 (62.4%) participants (63.5% in males and 61.9% in females). The mean SAS-SV score for the potential high-risk group was 43.18 ± 7.59 (42.33 ± 7.85 in males and 43.53 ± 7.45 in females). These alarming results warrant validation and intervention. In comparison, our results are different from those reported in China: 29.8% (17), South Korea: 24.8% (25), Spain: 12.8% (18), Belgium: 21.5% (18), Switzerland: 16.9% (21), but comparable to Lebanon: 44.6% (19), Morocco: 55.8% (30), and Saudi Arabia: 71.9% (20). All previously mentioned studies used the same assessment scale; SAS-SV. Interestingly, another study in Jordan conducted before the COVID-19 pandemic that used a different assessment scale and different cut-offs (39) reported addiction prevalence of 59.8%, compared to 27.2% in Saudi Arabia, 17.3% in Sudan, and 8.6 in Yemen, re-enforcing our findings of mobile phone overuse in Jordan.

The high prevalence of smartphone usage among the students is alarming and raises warning flags on the high risk of excessive use among Jordanians in general and during the quarantine in particular. Depression and anxiety are among the potential contributors to increased addiction to smartphones (40), factors which also increased under quarantine conditions (6, 41). A gender-based effect of mobile phone addiction was reported previously, with the prevalence of females showing more addictive symptoms and reporting more intensive use than males (39, 42–45), agreeing with our findings. Furthermore, a significant association between addiction levels and students' majors was observed in previous research; humanities, but not scientific and medical studies, were more commonly associated with smartphone addiction (39, 46–48). This contradicts our findings. Relying on smartphones for distance learning is more common in scientific/medical majors than humanities, which rely more on hard copy. Finally, although a few studies have demonstrated an association between academic performance and mobile addiction (49–51), no significance was reported in this study (*p*-value: 0.11).

Whether this can be classified as an addiction or overuse is still debatable (52). Panova et al. argue that the strict definition of addiction is not fulfilled in smartphone overuse. Smartphone soveruse is not associated with significant functional, financial or physical impairment. Besides, an increase in smartphone use is not equivalent to tolerance; nowadays, smartphone use is a normalized part of everyday life in many societies, even when engaged with very frequently (52). This is precisely what the students encountered during the quarantine. The dependence on distance learning, the substitution of hardcopy books and journal references with softcopies, affluence, and affordable free applications all helped direct the students toward smartphone overuse.

The study's limitations include the dependence on self-reporting of the use of smartphones, which might be associated with recall bias, thus under- or over-estimation. In addition, all students were from the same university, which might be associated with selection bias. However, the large number of participants (6,157), spread over various economic sectors, is an accurate reflection of Jordanian society, rendering the results generalizable. Another limitation is the potential selection bias resulted from having around 70% of female participants. More balanced selection criteria would be better to apply. However, this factor was controlled in the logistic regression model. Furthermore, increasing the reliance on remote learning during the imposed quarantine might be associated with the overuse of smartphones. The study should be repeated outside the quarantine period to give a better insight into the magnitude and the socio-cultural factors related to smartphone overuse.

## Conclusions and Recommendations

Quarantine is a stressful situation with several challenges, casting its shadow over routine life. No previous study has assessed the relationship between quarantine and smartphone addiction levels during the quarantine period. Female gender, urban areas, apartment quarantine, higher income, and scientific and medical majors had higher and significant overuse scores. The SAS-SV scores are higher than previously reported scores for other countries, although they are comparable to other countries in the region (39). Whether an addiction or overuse, the high scores and prevalence reported are alarming and indicate the severity of smartphone dependence among Jordanian university students during the quarantine. A repeat questionnaire on a comparable study population with follow-up interventions is warranted.

## Data Availability Statement

The raw data supporting the conclusions of this article will be made available by the authors, without undue reservation.

## Ethics Statement

The studies involving human participants were reviewed and approved by Institutional Review Board and the Research Ethics Committee at the University of Jordan. The ethics committee waived the requirement of written informed consent for participation.

## Author Contributions

HS conceived the idea, performed the analysis, and wrote the manuscript. RA performed the pre-processing and part of the statistical analysis and figures. HK performed part of the statistical analysis. AA, NS, and SA-S contributed to the literature search. MA-H co-wrote the manuscript and helped in designing the study. All authors contributed to the article and approved the submitted version.

## Conflict of Interest

The authors declare that the research was conducted in the absence of any commercial or financial relationships that could be construed as a potential conflict of interest.

## Publisher's Note

All claims expressed in this article are solely those of the authors and do not necessarily represent those of their affiliated organizations, or those of the publisher, the editors and the reviewers. Any product that may be evaluated in this article, or claim that may be made by its manufacturer, is not guaranteed or endorsed by the publisher.
